# Positive effects of dietary fiber from sweet potato [*Ipomoea batatas* (L.) Lam.] peels by different extraction methods on human fecal microbiota *in vitro* fermentation

**DOI:** 10.3389/fnut.2022.986667

**Published:** 2022-09-07

**Authors:** Yan Cao, Baoming Tian, Zhiguo Zhang, Kai Yang, Ming Cai, Weiwei Hu, Yang Guo, Qile Xia, Weicheng Wu

**Affiliations:** ^1^State Key Laboratory for Managing Biotic and Chemical Threats to the Quality and Safety of Agro-Products, Food Science Institute, Zhejiang Academy of Agricultural Sciences, Hangzhou, China; ^2^Food Natural Product and Nutritional Health Research Center, College of Food Science and Technology, Zhejiang University of Technology, Huzhou, China

**Keywords:** sweet potato peels, dietary fiber, *in vitro* fermentation, gut microbiota, short-chain fatty acids

## Abstract

The purpose of this study was to compare the effects of sweet potato peels dietary fiber obtained by different extraction methods on intestinal health. Specifically, four different dietary fibers were extracted by hot water, microwave, ultrasonic and subcritical water methods. And the prebiotics effects of sweet potato peels dietary fibers were explored in an *in vitro* fermentation model, by determining intestinal gas content, short-chain fatty acid content, pH, ammonia content and the gut microbiota composition. The results showed that dietary fiber obtained by four different extraction methods could be utilized by GM and improve human health by increasing the abundance of beneficial bacteria (e.g., *Bifidobacterium, Faecalibacterium*, and *Prevotella*) and reducing the abundance of harmful bacteria (e.g., *Proteobacteria, Romboutsia* and *Dorea*), enhancing the relative abundance of SCFA-producing bacteria, promoting the production of short-chain fatty acids, reducing intestinal pH from 6.89 to 4.63 and ammonia. Among them, dietary fiber extracted by ultrasound is better than the other three extraction methods. This study suggests that all the four different extraction methods are available for sweet potato peels dietary fiber, and the extracted dietary fiber could be served as potential functional foods with great development value. In addition, it is beneficial to reduce the environmental pollution of sweet potato peels and improve the high-value processing and utilization of sweet potato by-products.

## Introduction

As the core part of intestinal microecology, the gut microbiota (GM) is closely related to human health (1). Maintaining the balance of GM has a variety of positive effects, such as enhancing immunity, reducing blood lipids, anti-aging and anti-oxidation (2). The potential contribution of GM to human health has attracted widespread attention over the last decade. Studies have shown that some beneficial microbiota in the intestinal tract and its metabolites (e.g., short-chain fatty acids, SCFAs) can regulate intestinal discomfort and promote human health (3). The GM is related to a series of health problems, which are closely associated with dietary factors, and diet can influence host metabolism and the incidence of metabolic disorders by regulating the GM composition (4). In recent years, dietary regulation of GM has received extensive attention, and dietary intervention has become a commonly used intervention. As a new type of nutrient, dietary fiber can maintain human health by regulating the structure of GM, and has been widely used in the field of functional food (5). Dietary effects on the GM play key roles in the pathophysiology of inflammatory disorders, metabolic syndrome, obesity, and behavioral dysregulation (6). Dietary fiber is a carbohydrate polymer with 10 or more degrees of polymerization. There are two types of dietary fiber, water-soluble dietary fiber (SDF) and water-insoluble dietary fiber (IDF), according to their solubility in water (7). IDF may influence microbiota metabolites, which in turn are involved in regulating metabolic, immune, behavioral, and neurobiological outcomes (6). Because dietary fiber has many benefits, such as increasing satiety, relieving constipation, delaying the rise of blood sugar, reducing serum cholesterol, it has been widely used in food production (8). The recommended daily intake of dietary fiber is 50 g, but the amount should be determined by the individual. Dietary fiber is mainly divided into plant dietary fiber, animal dietary fiber, algae dietary fiber, microbial fiber and so on. Plant dietary fiber is the most studied type of dietary fiber because of its high yield, easy access and high nutritional value (9). Different extraction methods may not only affect the yield of dietary fiber, but also influence the functional activity of dietary fiber (10). With the development of industry, more and more extraction methods have been developed in the extraction of dietary fiber. Wakita et al. (11) used subcritical water to extract dietary fiber from soybean and investigated its effect on angiotensinogen I in rats. Song et al. (12) obtained bamboo shoot dietary fiber by enzymatic extraction, and further explored its related characteristics (8).

Sweet potato, *Ipomoea batatas* (L.) Lam., has wide production geography, from 40° north to 32° south latitude of the globe, and it is cultivated in 114 countries (13). It is a kind of natural nourishing food with complete nutrition, mainly growing in tropical and subtropical areas. Sweet potato contains protein, polysaccharide, fat, amino acids, carotene, vitamin and other nutrients. It plays an important role in promoting the activity of brain cells, enhancing the ability of resisting disease, improving the immune function and delaying the aging of the body (14). Sweet potato roots have high nutritional value and sensory versatility in terms of taste, texture, and flesh color (white, cream, yellow, orange, purple). At present, sweet potato processing products are mainly focused on sweet potato starch, sweet potato alcohol, sweet potato glucose and so on (15). There are few studies on the SDF of sweet potato peels. Dietary fiber has been shown to have a number of important associations with the development and management of various diseases and with mortality in epidemiological and interventional studies (16). Sweet potato processing industry had integrated a relatively complete supply chain. However, the main issue related with sweet potato processing industry is about the by-products. In order to make better use of sweet potato by-products, some researchers used response surface methodology to optimize the extraction process of soluble dietary fiber from steam explosion modified sweet potato residue (17). In addition, some researchers also extracted dietary fiber from the residues of 10 varieties of sweet potatoes. The approximate composition, chemical composition, monosaccharide composition and physicochemical properties of 10 kinds of sweet potato residue dietary fibers were further studied (18). In addition to sweet potato residue, sweet potato peel also contains a lot of fiber and flavonoids polyphenols. Based on previous research, the dietary fibers from the sweet potato residue, a previously little utilized industrial by-product showed its effects in promoting the abundance of beneficial gut microbiota species and short-chain fatty acids, and at the same time, improving the balance of gut bacteria and intestinal physiology (19). Therefore, it is an ideal candidate material for extracting functional dietary fiber.

In this study, four kinds of dietary fiber from sweet potato peel were obtained by hot water extraction (HWE), ultrasonic assisted extraction (UE), subcritical water extraction (SWE) and microwave assisted extraction (ME). The effects of sweet potato peel SDF obtained by four extraction techniques on intestinal fermentation were investigated by an *in vitro* simulated intestinal fermentation model. Four kinds of SDF extracted were used as carbon source, inulin as positive control group (INU) and inulin without carbon source as blank control group. After 24 h fermentation, the intestinal flora abundance, SCFA content, gas production, pH value, ammonia content, reducing sugar content and degradation rate were evaluated. The effects of dietary fiber extracted from sweet potato peel by different methods on intestinal microecology were further analyzed.

## Materials and methods

### Materials and chemicals

Sweet potato (Zhejiang sweet potato No. 13 variety) are washed, sliced, dried at 50°C until moisture content is below 5%, crushed, sifted through 100 mesh and moisture-proof packaged). Peptone, beef extract and yeast extract were purchased from Beijing Shuangxuan Microbial Culture Medium Products Factory (Beijing, China). Acetic acid (100%), propionic acid (99%), butyric acid (99%), isobutyric acid (99%), valeric acid (90%), and isovaleric acid (99%) were purchased from Sigma Corporation (USA). The enzyme-linked glucose assay kit was purchased from Suzhou Comin Biotechnology Co., Ltd. (Suzhou, China). All other chemical components are of analytical grade.

### Preparation of soluble dietary fiber from sweet potato

**Figure 1:**
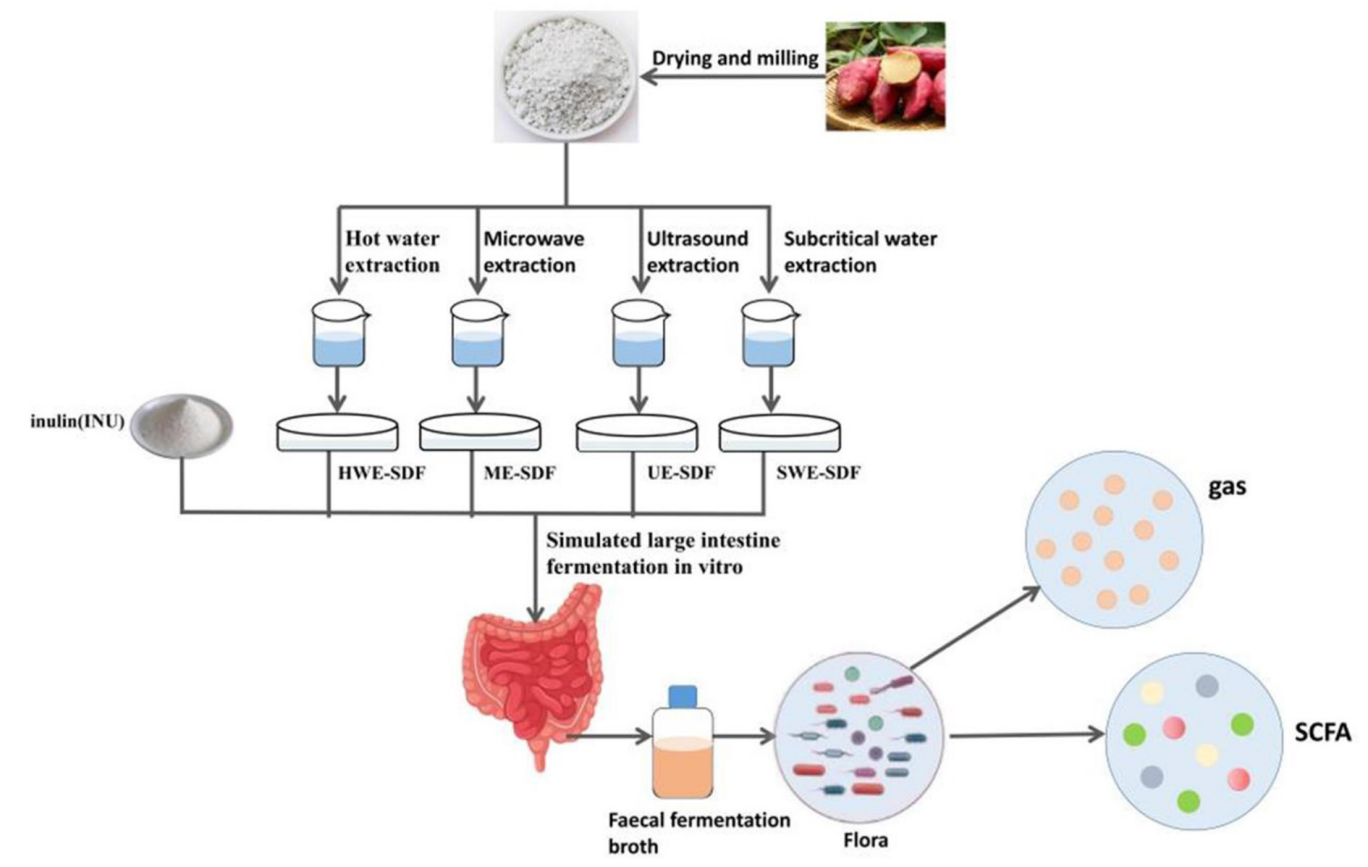
The schematic chart of extracting dietary fiber from sweet potato peels and simulating intestinal fermentation.

The sweet potato flour was extracted by four methods. A certain proportion of distilled water was added into sweet potato peels powder and fully stirred until the mixture was uniform, which was extracted by different physical methods. After the extraction, it was cooled to room temperature, and the pH value was adjusted to 6.0. The high-temperature-resistant α-amylase of 2% dry weight of sweet potato peel powder was added and heated in a water bath at 95°C for 30 min, then cooled to room temperature and adjusted to pH 4.5 (20). Starch glucosidase of 1% dry weight of sweet potato skin powder was added and heated in a 60°C water bath for 30 min, then cooled to room temperature and adjusted the pH value to 7.0. Then 0.1% dry weight trypsin of sweet potato peel powder was added and heated in a 60°C water bath for 30 min. After taking them out, the extracts were cooled to room temperature and centrifuged at 4,000 r/min for 10 min. The supernatant was taken and added with 95% ethanol of 4 times volume overnight. The supernatant was removed and centrifuged at 4,000 r/min for 20 min. The centrifugal precipitate was removed and freeze-dried for 72 h, and then crushed to obtain SDF (21). The extracted solution after enzyme treatment was directly added with 95% ethanol of 4 times volume overnight, and the supernatant was removed and centrifuged at 4,000 r/min for 20 min. The centrifugal precipitate was removed and freeze-dried for 72 h, and crushed to obtain the total dietary fiber (TDF) (22). The content and yield of TDF and SDF were determined and calculated by referring to the enzyme gravimetric method of Mccleary et al. (23). HWE: the ratio of solid to liquid was 1:20, the extraction time was 3 h, the extraction temperature was 60°C, and the SDF and TDF yield were 1.20 and 21.72%, respectively. UE: the ratio of solid to liquid was 1:30, the extraction time was 30 min, the extraction temperature was 70°C, the extraction power was 400 W, and the SDF and TDF yield were 8.21 and 22.23%, respectively. SWE: the ratio of solid to liquid was 1:30, the extraction time was 30 min, the extraction temperature was 120°C, and the SDF and TDF yield were 10.43 and 24.68%, respectively. ME: the ratio of solid to liquid was 1:30, extraction time 6 min, extraction power 500 W, and the SDF and TDF yield were 7.74 and 21.84%, respectively. The scehmatic chart is shown in Figure 1.

### Preparation of medium and solution

Based on a previous method, the detailed operation procedure is as follows (3, 24).

1. Vitamin I solution: 40 ml of sterile water, 6 mg of *p*-aminobenzoic acid, 2 mg of cyanocobalamin, 30 mg of pyridoxamine, 10 mg of folic acid, 2 mg of vitamin H, stored at −40°C.
2. Vitamin II solution: 1 ml of sterile water, 5 mg of riboflavin, 5 mg of thiamine, and stored at −40°C.
3. PBS solution: sterile water 1 L, Na_2_HPO_4_ 1.44 g, KH_2_PO_4_ 0.24 g, KCl 0.2 g, NaCl 8 g, placed at room temperature.
4. Human stool processing buffer: 1 L of PBS solution, 100 μl of vitamin II solution, placed at room temperature.
5. Basal medium: sterile water 1.2 L, KH_2_PO_4_ 0.54 g, MgSO_4_·7H_2_O 0.108 g, CaCl_2_·6H_2_O 0.108 g, NaCl 1.08 g, l-cysteine hydrochloride 1.2 g, heme 2.4 ml, Yeast extract 3 g, tryptone 12 g, vitamin I solution 240 μl, stored at room temperature.
6. Fermentation medium: SDF prepared by different extraction methods was added to the basal medium at a concentration of 0.8% as the sample medium; the commercially available inulin was used as the positive control; the blank control was the basal medium.
7. Sterilize the basal medium at high temperature and cool it, use a nitrogen blower to fill the fermentation flask with nitrogen, then quickly pump 5 ml of the basal medium and seal it for storage.

### Preparation of human fecal fluid

Feces were collected from 4 healthy male and 4 healthy female volunteers (who lived in Hangzhou, Zhejiang Province, aged 21–40 years old, had a normal diet, and did not take or inject antibiotics 3 three months. This study was approved by the Ethics of Zhejiang Academy of Agricultural Sciences Committee and all volunteers obtained informed, written consent). Take 1 g of feces and put it into a membrane-filtered fecal collection bottle, seal it, put it into a feces processing apparatus, pump 10 ml of PBS solution, vortex and let it stand. The fecal diluent gradually seeps out through the membrane filtration in the bottle, and 5 ml of the filtered diluent is drawn with a syringe for use (25).

Inoculation: Use a syringe to draw 500 μl of fecal filtrate from a single person and inject it into 6 kinds of fermentation bottles, shake well, label, and ferment in a 37°C incubator.

### Determination of gases

The fermentation flask was taken out after 24 h of fermentation, and a barometer (Thommen gas pressure measuring instrument, Tianjin Celiss Automation Technology Co., Ltd., Tianjin, China) was used to pierce the fermentation flask and record the air pressure value (25).

### Determination of short-chain fatty acids

Use a syringe to draw 650 μl of the fermentation broth into a 1 ml centrifuge tube, and freeze and centrifuge at 20,000 r/min. Then use a pipette to pipette 500 μl of the centrifuged supernatant into a 1.5 ml centrifuge tube, add 100 μl of crotonic acid, vortex for 3 min, and store at −40°C for later use. The concentration of SCFA in the fermentation flask was analyzed by gas chromatography (GC), which mainly included the content of acetic acid, propionic acid and butyric acid. Chromatograph model: GC-2010 Plus (Shimadzu, Japan), column type: DB-FFAP125-3237, size: 30 m × 0.53 mm × 0.5 μm, nitrogen as the load gas, flow rate 14.4 ml/min. Initial temperature: 100°C for 0.5 min, then increased to 180°C at 8°C/min and held for 1 min, and finally increased to 200°C at 20°C/min and held for 5 min. The detector and injection port temperatures were controlled to be 240°C and 200°C, respectively. Set the nitrogen, hydrogen, and air flow rates to 20, 40, and 400 ml/min, respectively. The injection volume was 1.0 μl, and the analysis time was 17.5 min (26).

### Determination of pH value

The fermentation broth after fermentation was taken, refrigerated and centrifuged at 20,000 r/min, and the pH value was measured and recorded using a pH detector (PHS-3C, Hangzhou Aolilong Instrument Co., Ltd., Hangzhou, China). Ammonia content was measured and recorded using an ammonia calibration standard kit (Meikang Biotechnology Co., Ltd., Ningbo, Zhejiang) (27).

### Determination of reducing sugar content after fermentation

Determination of reducing sugar content by DNS reagent (28). A standard curve was prepared with 1 mg/ml glucose standard solution. Take 2 ml of the centrifuged fermentation supernatant, add 2 ml of DNS reagent, boil in water for 10 min, cool to room temperature, dilute to 25 ml, mix well, measure the absorbance at 540 nm, and repeat three times in parallel.

### Determination of dietary fiber degradation rate by thin layer chromatography

Developing agent: formic acid: *n*-butanol: water (volume) = 6:4:1; Color developer: 1.8 g lichenol dissolved in 50 ml water, add 750 ml ethanol, ice bath and slowly add 100 ml concentrated sulfuric acid; Spotting: Use a pipette to suck up 2 μl of the supernatant obtained by centrifugation after fermentation, place them on the thin layer chromatography (TLC) silica gel plate showing the drawn lines in sequence, and dry them with hot air; Development: Lean the silica gel plate on which the sample has been placed in a closed chromatographic cylinder. The height of the developer page is 1 cm away from the sample point. After the developer is inclined upward to the top of the silica gel plate, take out the hot air and dry it. Color development: evenly spray the color developer onto the silica gel plate and dry it with hot air. Analysis: The colored silica gel plate was placed in a TLC oligosaccharide analyzer to analyze its degree of degradation (29).

### DNA extraction and high-throughput sequencing of gut microbiota and bioinformatic analysis

According to statistical requirements, five samples were randomly selected after fecal fermentation. The fermentation broth was centrifuged, the pellet was collected after centrifugation, and the DNA of the sample was extracted using a DNA kit (Omega Bio-tek, Norcross, GA, U.S.). After the sample DNA was extracted, the extracted genomic DNA was detected by 1% agarose gel electrophoresis. Subsequent PCR amplification: according to the designated sequencing region, specific primers with barcode are synthesized (each sample is repeated three times, the PCR products of the same sample are mixed, and they are detected by 2% agarose gel electrophoresis. The variable regions V3–V4 of the bacterial 16S rRNA genes were performed using the broadly conserved PCR forward primers 338F (5 –ACTCCTACGGGAGGCAGCAG – 3) and reverse primer 806R (5 – GGACTACHVGGGTWTCTAAT – 3) by an ABI GeneAmp^®^ 9700 PCR thermocycler (ABI, CA, USA). Subsequently, PCR products were recovered with AxyPrepDNA gel recovery kit, eluted with Tris-HCl and detected by 2% agarose electrophoresis). According to the preliminary quantitative results of electrophoresis, the PCR products were detected and quantified by QuantiFluor™-ST blue fluorescence quantitative system, and then the corresponding proportions were mixed according to the sequencing amount of each sample. Finally, Illumina PE library construction and Illumina PE sequencing were performed, and the obtained data were subjected to corresponding biological analysis (30).

Purified amplicons were pooled in equimolar and paired-end sequenced on NovaSeq PE250 platform (Illumina, San Diego, USA) according to the standard protocols by Majorbio Bio-Pharm Technology Co. Ltd. (Shanghai, China). The raw 16S rRNA gene sequencing reads were demultiplexed, quality-filtered by fastp version 0.20.0 and merged by FLASH version 1.2.7 with the following criteria: (i) the 300 bp reads were truncated at any site receiving an average quality score of <20 over a 50 bp sliding window, and the truncated reads shorter than 50 bp were discarded, reads containing ambiguous characters were also discarded; (ii) only overlapping sequences longer than 10 bp were assembled according to their overlapped sequence. The maximum mismatch ratio of overlap region is 0.2. Reads that could not be assembled were discarded; (iii) Samples were distinguished according to the barcode and primers, and the sequence direction was adjusted, exact barcode matching, two nucleotide mismatch in primer matching. Operational taxonomic units (OTUs) with 97% similarity cutoff were clustered using UPARSE version 7.1, and chimeric sequences were identified and removed. The taxonomy of each OTU representative sequence was analyzed by RDP Classifier version 2.2 against the 16S rRNA database using confidence threshold of 0.7.

### Statistical analysis

The experimental data of each group were measured three times in parallel and expressed as mean ± standard deviation. Data analysis was performed using SPSS 20.0 (SPSS Inc., Chicago, Illinois, USA), and *p* < 0.05 was considered to be statistically significant. Graphing was performed with Origin 2018 software (OriginLab, USA).

## Results and discussion

### Effects of dietary fiber from sweet potato peels *in vitro* fermentation on air pressure

**Figure 2:**
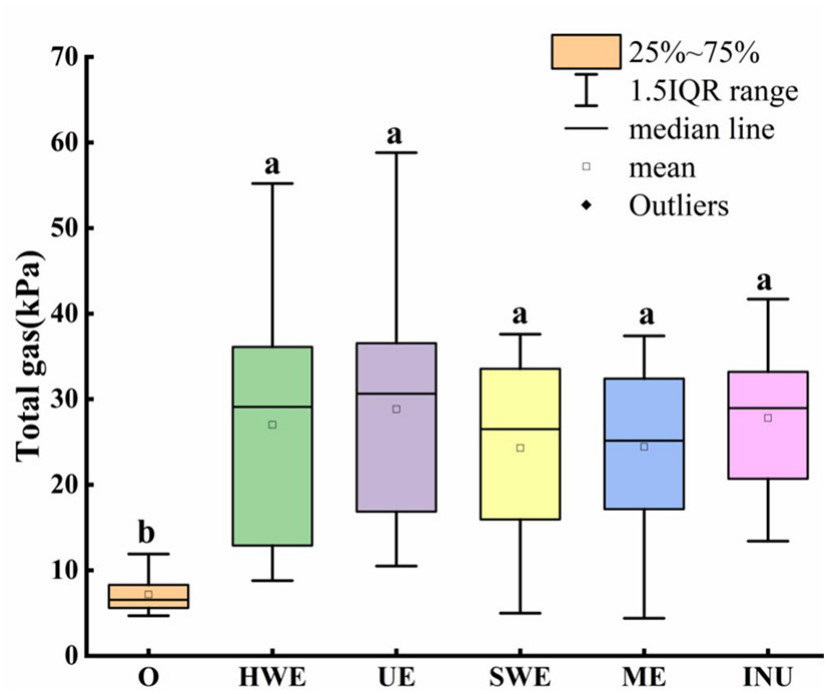
Gas production of *in vitro* fermentation samples. O stands for blank control group. HWE, UE, SWE, and ME stand for dietary fiber extracted with hot water, ultrasonic assisted, subcritical water, and microwave assisted methods, respectively. INU stands for inulin. Different letters a and b indicate significant differences *(p* < 0.05), the same letters indicate that the differences are not significant (*p* > 0.05).

The gas in the intestines is closely related to the health of the human body. After the dietary fiber enters the human body, it will be used by the intestinal flora to generate gas, thus affecting human health (31, 32). Gas production in the intestine is shown in Figure 2. The median values of UE, HWE and INU are skewed, so only the mean value cannot accurately compare the pros and cons of the data. Compared with the blank control group, it was found that the gas production increased significantly after adding carbon source (*p* < 0.05), so it was proved that the dietary fiber could be decomposed and utilized by the intestinal flora to generate gas. Further analysis of the median value of each group showed that the UE group had the largest number of people with higher gas production, which was higher than that by other extraction methods. An analysis of the quartiles shows that UE, HWE and INU are generally similar. Although the dispersion is slightly larger than that of INU, the overall gas production of HWE and UE is close to that of commercially available INU, and both have good population adaptability. In addition, SWE and ME show lower gas production, so they are more universal. Further observation of the dispersion degree of each box shows that, except for the blank control group, the dispersion degree of INU is the lowest, indicating that INU has a relatively stable adaptability to the gas production of different human intestinal flora. Compared with the commercially available INU, the SDF obtained by the four extraction methods has an acceptable impact on human gas production, does not cause obvious flatulence, and has good potential development value.

### Effects of dietary fiber from sweet potato peels *in vitro* fermentation on short-chain fatty acid production

**Figure 3:**
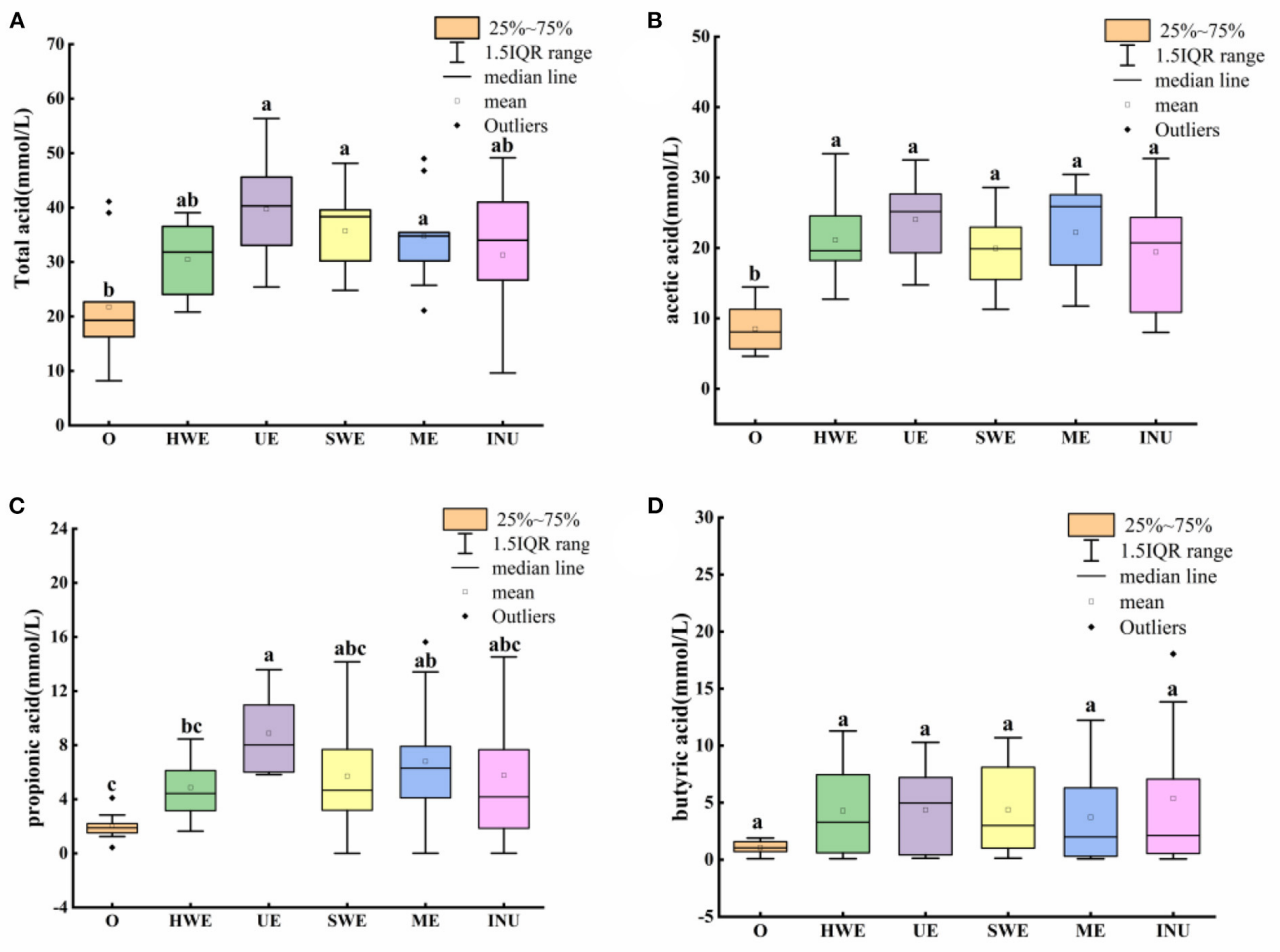
Short-chain fatty acid content in *in vitro* fermentation samples. O stands for blank control group. HWE, UE, SWE, and ME stand for dietary fiber extracted with hot water, ultrasonic assisted, subcritical water, and microwave assisted methods, respectively. INU stands for inulin. Different letters a, b and c indicate significant differences (*p* < 0.05), the same letters indicate that the differences are not significant (*p* > 0.05). **(A)** Total acid content, **(B)** acetic acid content, **(C)** propionic acid content, and **(D)** butyric acid content.

Short-chain fatty acids are saturated fatty acids with a chain length of 1–6 carbon atoms, which are the main products of dietary fiber fermentation in the intestine, mainly including acetic acid, propionic acid, butyric acid, etc. (33). As shown in Figure 3A, combining the mean and outliers, the individual differences in the content of total short-chain fatty acids are large, so the mean value cannot reflect the influence trend well. From the median value, UE has the most significant improvement in total acid (*p* < 0.05), followed by SWE (*p* < 0.05) and ME (*p* < 0.05). Although there were outliers in the ME data, and the median value was close to INU, the interquartile range was smaller than that of commercially available INU, indicating ME has smaller individual differences and more stable fermentation performance than INU. From the perspective of promoting SCFA content, the UE group was the best among the four extraction methods. The promotion effect of SDF obtained by the four extraction methods on SCFA is close to or stronger than that of commercially available INU, which has great potential for development.

Acetic acid, as the most abundant SCFAs, can be directly absorbed by the blood for energy supply in the brain, heart and other parts, and plays an important role in human health. *Lactobacillus, Provotella, Bifidobacterium, Luminococcus*, and *Akkermansia* are acetic acid-producing bacteria, they can control human appetite by producing acetic acid, and can also promote the growth of butyric acid-producing bacteria, thereby maintaining gut health (34, 35). As shown in Figure 3B, after adding carbon source, the acetic acid content in each group increased significantly (*p* < 0.05). The observed mean found that the HWE was higher than that of the SWE. However, combined with the median value, it is found that the median value of HWE is skewed and close to that of SWE. Although the improvement of UE and ME is close, the number of people whose improvement is in the middle and high level of ME is larger than that of UE. Combining quartiles and medians, both UE and ME had better effects on acetic acid content than commercially available INU. And the data trend is more concentrated, which means that the population adaptability is more stable.

Propionic acid, another product of bacterial breakdown of dietary fiber, has many health benefits. Bacteroidetes and phylum family bacteria are the main producers of propionic acid, which can regulate appetite, resist inflammation, and help prevent cancer by producing propionic acid (36). As shown in Figure 3C, the effect of ME on the content of propionic acid showed abnormal values, which may be due to the difference between individuals. From the median value, UE has the most distinct increase in propionic acid content (*p* < 0.05). The content of propionic acid in ME group was higher than that in HWE group (*p* < 0.05) and INU group. Analyzing the quartiles, SWE and ME were close overall. However, the ME group has more people with the lift in the middle and high levels than SWE, and the population difference of ME is the smallest after removing outliers.

Although the content of butyric acid in the intestine is very few, it is essential to human health. *Faecalibacterium prausnitzii* and *Roseburia* are butyrate-producing bacteria that not only help prevent leaky gut, fight inflammation and cancer by producing butyrate, but also help fight neurodegeneration like Alzheimer's and Parkinson's illness, as well as mental health disorders and autism (37). As shown in Figure 3D, comparing the mean values, it is found that HWE, UE and SWE are not significantly different (*p* > 0.05). However, from the perspective of the median value, UE has the best effect on increasing butyric acid, and can effectively promote the increase of butyric acid content. According to a previous study, dietary fiber from sweet potato residue could increase the intestinal SCFAs content in rats, which was further confirmed by the decrease of pH (19).

### Effects of dietary fiber from sweet potato peels *in vitro* fermentation on pH and ammonia content

After the dietary fiber enters the intestinal tract, it will be decomposed and utilized by the intestinal flora to produce short-chain fatty acids, which further regulates the intestinal pH. The stability of intestinal pH is essential to human health, and a lower pH value can reduce the growth and reproduction of harmful bacteria in the intestinal flora (38). As shown in Table 1, compared with the blank control, the pH of the dietary fiber group was significantly decreased (*p* < 0.05). The order of pH value from low to high is INU < ME ≤ UE < SWE < HWE.

**Table 1:** Analysis of pH and ammonia content of *in vitro* fermentation samples.

**Extraction method**	**pH value**	**Ammonia content (μmol/L)**
Blank	6.89 ± 0.37a	1,849.94 ± 385.82a
HE	5.28 ± 0.57b	1,287.81 ± 290.25b
UE	4.83 ± 0.49cd	709.21 ± 149.43d
SE	4.87 ± 0.32c	764.72 ± 176.61c
ME	4.63 ± 0.33cd	679.73 ± 166.91e
INU	4.31 ± 0.45d	580.11 ± 128.50g

HWE, UE, SWE, and ME stand for dietary fiber extracted with hot water, ultrasonic assisted, subcritical water, and microwave assisted methods, respectively. INU stands for inulin. Different letters a, b and c indicate significant differences (p < 0.05), and the same letters indicate that the difference is not significant (p > 0.05).

Ammonia in the gut is the main source of blood ammonia. Intestinal bacteria have urease, which can hydrolyze urea into CO_2_ and NH_3_. Studies have shown that excessive levels of ammonia can have harmful effects on the human body. Among the four groups supplemented with dietary fiber, ME had the most significant reduction in ammonia content, and the effect was closest to that of commercially available. Ammonia content affects intestinal pH, and the changes in pH and ammonia content in this experiment are consistent with the results of Chen et al. (39).

### Analysis of reducing sugar proportion and degradation rate

During *in vitro* fermentation, the breaking of glycosidic bonds leads to exposure of more reducing ends as dietary fiber is consumed and utilized by the GM. Therefore, the utilization of carbon sources can be inferred from the proportion of reducing sugars (27). As shown in Table 2, among the SDF obtained by the four extraction methods, UE has the highest degradation rate in the human *in vitro* fermentation simulation, and has no significant difference with the commercially available INU. In addition, among the SDFs obtained by the four extraction methods, UE had the most significant effect on the ratio of reducing sugars to total sugars after fermentation (*p* < 0.05). From the order of significance analysis results, the ratio of reducing sugar to total sugar after fermentation was consistent with the degradation rate. This may be the reason that during *in vitro* fermentation, fecal microbiota use polysaccharide fermentation to degrade macromolecular long-chain polysaccharides into small-molecule sugars. After being degraded into small-molecule sugars, more reducing groups are exposed, thus increasing the proportion of reducing sugars after fermentation.

**Table 2:** Analysis of reducing sugar content and degradation rate of *in vitro* fermentation samples.

**Extraction method**	**Reducing sugar/total sugar (%)**	**Degradation rate (%)**
HE	43.74 ± 8.66d	62.17 ± 5.33d
UE	67.9 ± 4.21a	77.23 ± 4.69a
SE	58.34 ± 5.64b	74.41 ± 4.37b
ME	54.25 ± 6.48c	72.78 ± 5.45c
INU	68.35 ± 5.29a	80.52 ± 3.74a

HWE, UE, SWE, and ME stand for dietary fiber extracted with hot water, ultrasonic assisted, subcritical water, and microwave assisted methods, respectively. INU stands for inulin. Different letters a, b and c indicate significant differences (p < 0.05), the same letters indicate that the differences are not significant (p > 0.05).

### Effects of dietary fiber from sweet potato peels on α and β diversity of the fecal microbiota *in vitro* fermentation

#### α diversity

**Figure 4:**
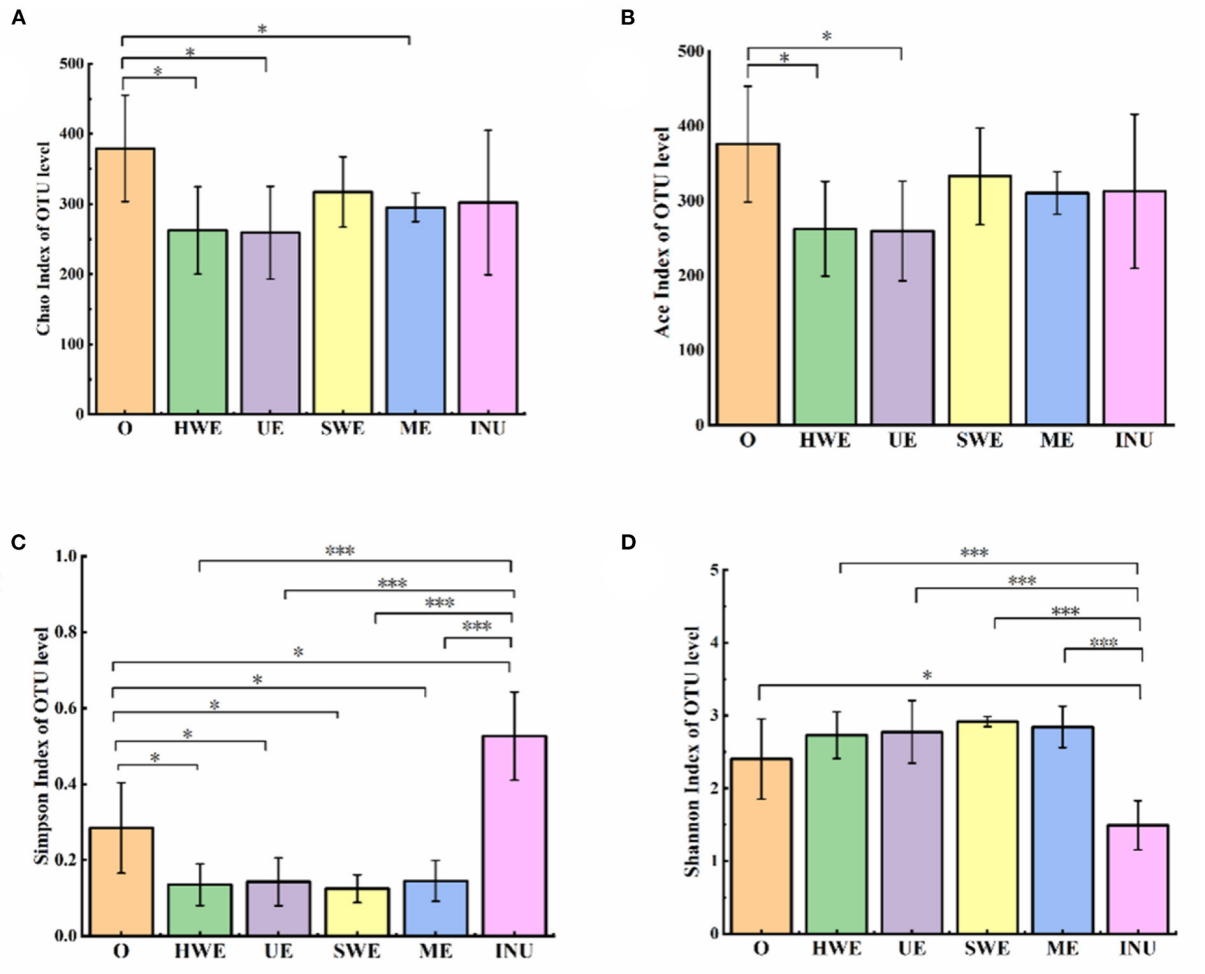
**(A-D)** Species richness and diversity analysis (*n* = 5). O stands for blank control group. HWE, UE, SWE, and ME stand for dietary fiber extracted with hot water, ultrasonic assisted, subcritical water, and microwave assisted methods, respectively. INU stands for inulin. *P <0.05, ***P < 0.001.

The indices of community diversity include the Simpson index and the Shannon index. The larger the Simpson index value, the lower the community diversity. In this analysis, the indexes reflecting community richness were Chao l index and ACE index. Shannon index and Simpson index were used to reflect community diversity. Compared with blank group, HWE and UE significantly reduced Chao1 and ACE index (*p* < 0.05), Chao1 index also decreased significantly in ME group (*p* < 0.05), indicating that HWE, UE and ME reduced fecal flora richness. As shown in the Figure 4, compared with the blank group, the Simpson index value in the INU group was significantly increased (*p* < 0.05), and the remaining groups were significantly decreased (*p* < 0.05). The Shannon index is also an index used to estimate the diversity of microorganisms in a sample. The larger the Shannon value, the higher the community diversity. As shown in the Figure 4, compared with the blank group, the Shannon value in the INU group decreased significantly, and the SDF groups increased significantly except for the HE group. Through analysis, we can conclude that the diversity of the mid-community decreased after adding INU, while the diversity of the colony increased after adding dietary fiber.

#### β diversity

**Figure 5:**
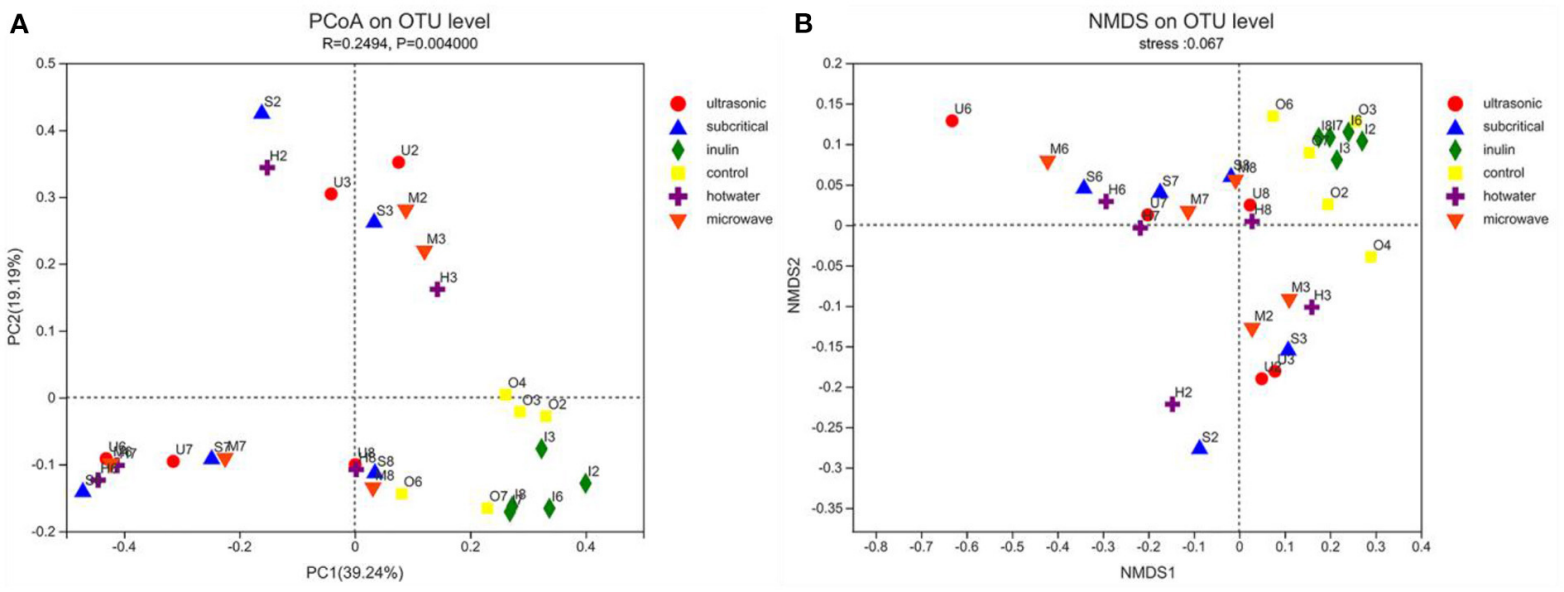
**(A,B)** The PCoA and NMDA plots show the β diversity of the fecal microbiota (*n* = 5). Control stands for blank control group. The hot water, ultrasonic, subcritical, and microwave stand for dietary fiber extracted with hot water, ultrasonic assisted, subcritical water, and microwave assisted methods, respectively.

β diversity analysis was performed to compare the magnitude of diversity differences between groups. Each point on the PCoA diagram represents an independent sample, and the distance between points reflects the diversity of similarity between samples. The results showed that the blank group and inulin could cluster effectively respectively, and the distance between them was relatively close (Figure 5A). These two groups were separated from the four kinds of extracted SDF groups, indicating that there were differences in the microbial community of the extracted fibers during treatment. The distribution of SDF extracted by the four physical methods was relatively scattered, and the distance between any two groups was relatively close, indicating that there were very similar species types between these groups. We further performed nonmetric dimensional scaling (NMDS) of weighted UniFrac analyses to investigate the potential differences. In accordance with the previous analysis, NMDS showed that the aggregation of the three groups was also clearly different (Figure 5B). NMDS data showed that the difference in aggregation was basically consistent with the results of PCoA. The blank group and inulin group were clustered together, and several dietary fiber groups were clustered together (Figure 5B).

Control stands for blank control group. The hot water, ultrasonic, subcritical, and microwave stand for dietary fiber extracted with hot water, ultrasonic assisted, subcritical water, and microwave assisted methods, respectively.

#### Difference in dominant microorganisms under different treatments

**Figure 6:**
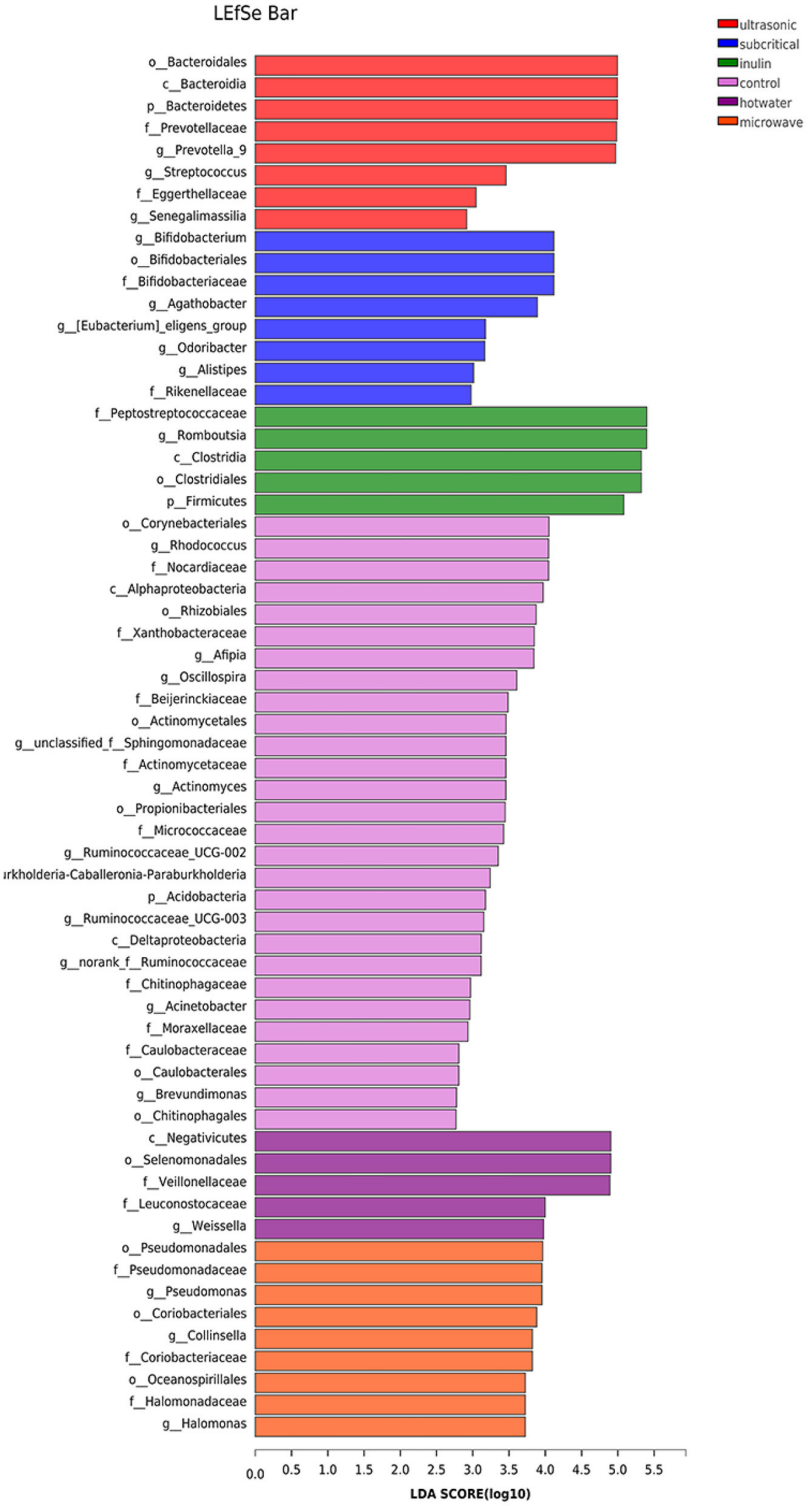
Bacterial taxa identified as differentially abundant between groups according to linear discriminant analysis effect size (LEfSe) (*n* = 5). Control stands for blank control group. The hot water, ultrasonic, subcritical, and microwave stand for dietary fiber extracted with hot water, ultrasonic assisted, subcritical water, and microwave assisted methods, respectively.

As shown in Figure 6, the analysis revealed 8, 8, 6, 28, 5 and 9 features that were significantly different in the UE, SEW, INU, blank, HWE, and ME groups, respectively. At the phylum level, the abundances of *Bacteroidetes* in the UE group, and *Acidobacteria* in the blank group were enriched and significantly different. At the genus level, the abundances of *Prevotella_9, Streptococcus*, and *Senegalimassilia* in the UE group; *Bifidobacterium, Agathobacter, [Eubacterium]_eligens_group, Odoribacter* and *Alistipes* in the SEW group; *Romboutsia* in the INU group; *Rhodococcus, Afipia, Oscillospira, unclassified_f__Sphingomonadaceae, Actinomyces, Ruminococcaceae_UCG-002, Burkholderia-Caballeronia-Paraburkholderia, Ruminococcaceae_UCG-003, norank_f__Ruminococcaceae, Acinetobacter*, and *Brevundimonas* in the blank group; *Weissella* in the HEW groups; and *Pseudomonas, Pseudomonas*, and *Halomonas* in the ME group were substantially different.

### Effects of dietary fiber from sweet potato peels on the fecal microbial taxonomic profiles *in vitro* fermentation

The balance of intestinal flora is closely related to human health. We can help the human body prevent diseases and improve human health by exploring the relationship between sweet potato dietary fiber and intestinal flora.

#### Phylum level

**Figure 7:**
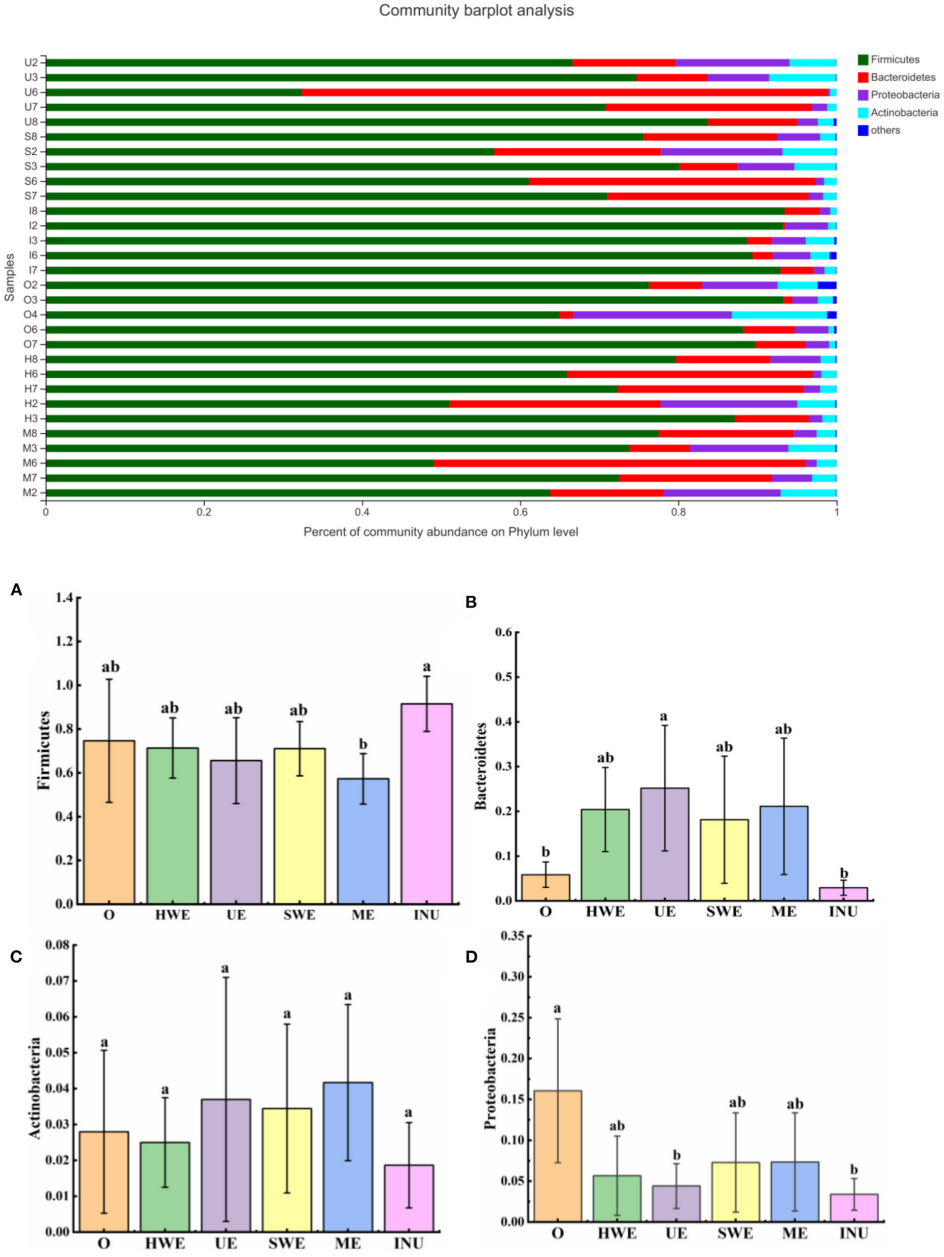
**(A-D)** Relative abundance of gut microbiota at the phylum level (*n* = 5). O stands for blank control group. HWE or H, UE or U, SWE or S, and ME or M stand for dietary fiber extracted with hot water, ultrasonic assisted, subcritical water, and microwave assisted methods, respectively. INU or I stand for inulin. Different letters a and b indicate significant differences (*p* < 0.05), the same letters indicate that the differences are not significant (*p* > 0.05).

The intestinal flora is classified on the phylum level, which is mainly divided into Firmicutes, Bacteroidota, Proteobacteria, and Actinobacteria. Some studies have found that Firmicutes can degrade glycans, thereby improving the utilization of glycans (40). As shown in Figure 7, the relative abundance of Firmicutes was analyzed, and it was found that except for the significant decrease in ME (*p* < 0.05), there was no significant change in other groups (*p* > 0.05). Some studies have shown that the value of Firmicutes/Bacteroides is related to obesity, but the specific proportional relationship has not yet been determined, and further research is needed (41). Bacteroides can increase relative abundance by degrading glycans. The relative abundance of Bacteroides was analyzed, and it was found that except the INU group, the relative abundance of each experimental group increased, and the relative abundance of bacteria in the UE group increased significantly (*p* < 0.05). The growth of Bacteroides in this experiment further verified that dietary fiber was degraded and utilized, and the UE group had the highest utilization rate. Actinobacteria is one of the four major bacterial phyla in the body, which can degrade complex polymers, and can also produce antimicrobial agents to help other bacteria and fungi in biological control. Statistics on the relative abundance of Actinobacteria showed that there was no significant change in the relative abundance of Actinobacteria in each experimental group (*p* > 0.05). There are both beneficial bacteria such as Bifidobacteria and some harmful bacteria in the actinomycete phylum. To further explore its function, in-depth analysis at the genus level is required (42). The relative abundance of Proteobacteria was counted, and it was found that the relative abundance of Proteobacteria in each experimental group and positive control group decreased, among which the UE group and INU group decreased significantly (*p* < 0.05). Through analysis, we can find that the relative abundance of harmful bacteria Proteobacteria can be reduced by adding dietary fiber, thereby improving intestinal health, among which UE has the best effect.

#### Genus level

**Figure 8:**
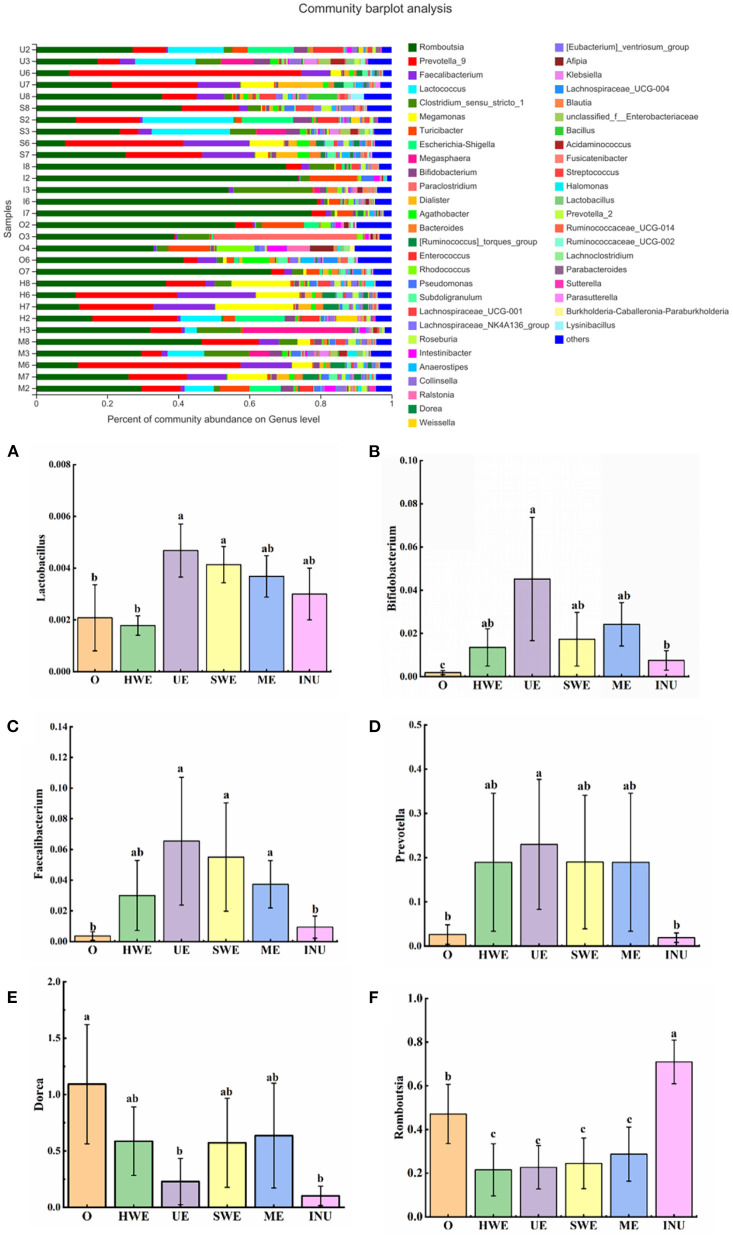
**(A-F)** Relative abundance of gut microbiota at the genus level (*n* = 5). O stands for blank control group. HWE or H, UE or U, SWE or S, and ME or M stand for dietary fiber extracted with hot water, ultrasonic assisted, subcritical water, and microwave assisted methods, respectively. INU or I stand for inulin. Different letters a, b and c indicate significant differences (*p* < 0.05), the same letters indicate that the differences are not significant (*p* > 0.05).

At the genus level, *Lactobacillus* and *Bifidobacterium* are typical intestinal beneficial bacteria. It can inhibit the growth of harmful intestinal bacteria by fermenting sugar substances to produce lactic acid, butyric acid, etc., thereby reducing the production of toxins (43). As indicated in Figure 8, statistical analysis of the relative abundance of Lactobacillus showed that compared with the blank group, the relative abundance of *Lactobacillus* in the UE group and the SWE group increased significantly (*p* < 0.05). Statistical analysis of the relative abundance of *Bifidobacterium* found that the relative abundance of *Bifidobacterium* in each group increased sdistinctly, and the UE group had the most significant increase (*p* < 0.05) (44). *Faecalibacterium*, a beneficial intestinal bacterium, can produce butyric acid and CO_2_ in the colon, and has anti-obesity and anti-inflammatory effects (45). Through the statistics of the relative abundance of *Faecalibacterium*, it was found that except the positive control group, the relative abundance of *Faecalibacterium* in each dietary fiber experimental group increased. Among them, UE group, SWE group and ME group increased significantly (*p* < 0.05). *Prevotella* in the human gut has a positive effect on gut health. It can improve glycogen storage capacity by improving glucose metabolism (46). Statistical analysis of *Prevotella* indicated that the relative abundance of *Prevotella* increased in all experimental groups except the positive control group. Among them, the UE group increased significantly (*p* < 0.05). *Dorea* is a bacterium found in human feces. Studies have shown that the abundance of Dorea is positively correlated with obesity, and its abundance has been found to be increased in prediabetic patients. Analysis of the bacterial abundance of *Dorea* found that compared with the blank group, the bacterial abundance of *Dorea* decreased in each experimental group, and that in the UE group and the INU group decreased significantly. *Romboutsia* is a colony associated with obesity. Shulzhenko et al. (47) found that *Romboutsia* flora exists in more than 80% of obese patients, suggesting that *Romboutsia* may be a microbial community prevalent in overweight people. The statistics of *Romboutsia* showed that, compared with the blank control group, the relative abundance of *Romboutsia* in all experimental groups except the INU group decreased significantly (*p* < 0.05). According to the flora analysis, we can conclude that adding dietary fiber can improve intestinal health by increasing the relative abundance of beneficial bacteria such as *Bifidobacterium, Faecalibacterium*, and *Prevotella*, and reducing the relative abundance of harmful bacteria such as *Romboutsia* and *Dorea*. Among them, the dietary fiber UE obtained by ultrasonic extraction has the most significant effect.

The relationship between short-chain fatty acids and flora abundance was analyzed to explore the regulatory mechanism of dietary fiber on intestinal microecological balance. By analyzing the gut microbiota at the phylum and genus levels, we can know that the increase in the abundance of *Bifidobacterium, Lactobacillus*, and *Prevotella* in the gut is related to the increase in the content of intestinal acetate. The increased abundance of *Bacteroidetes* was associated with increased propionic acid content. Increased abundance of *Facecalibacterium* was associated with increased butyric acid content. At present, the specific regulation function of short-chain fatty acids on the intestine has been studied a lot. Through this experiment, we can know that adding dietary fiber from sweet potato peel can promote the production of short-chain fatty acids, thereby maintaining human health. Among them, UE has the best effect. Other IDF from other parts of the sweet potato showed a similar trend. IDF from sweet potato dregs was effective on the intestinal flora of mice, and the middle dose (1.5 g/kg.day) group showed better effects than low dose (1.5 g/kg.day) and high dose (1.5 g/kg.day). The middle dose IDF from sweet potato dregs increase the abundance of *Lactobacillus* and *Faecalibaculum* (beneficial bacteria, which maintain the intestinal flora balance and have anti-inflammatory effects) (48). Other findings also support the utilization of dietary fiber from sweet potato residue in the development of potentially prebiotic food products for improving intestinal health. Dietary fiber from sweet potato residue mediated a significant increase in the concentrations of *Bifidobacterium* and *Lactobacillus*, whereas induced a significant decrease of *Enterobacillus* and *Bacteroides* (19).

## Conclusions

In this study, four different dietary fibers were obtained by hot water extraction, microwave extraction, ultrasonic extraction and subcritical water extraction. In addition, *in vitro* in-depth simulated fermentation experiments were carried out. By comparing gas, short-chain fatty acids, pH, ammonia and flora abundance, the regulation effect of dietary fiber on intestinal flora was deeply explored. The results showed that, compared with the blank group, the four experimental groups supplemented with sweet potato dietary fiber had moderate gas content, increased SCFA contents, decreased pH and ammonia contents. In addition, the abundance of beneficial bacteria in the gut increased and the abundance of harmful bacteria decreased after dietary fiber supplementation. Through analysis, we can conclude that adding sweet potato dietary fiber can improve the health of intestinal microecology. Among them, the sweet potato dietary fiber extracted by ultrasonic has the best improvement effect on the overall intestinal microecological health.

## Data availability statement

The datasets presented in this study can be found in online repositories. The names of the repository/repositories and accession number(s) can be found below: https://www.ncbi.nlm.nih.gov/bioproject/PRJNA856050 BioProject ID: PRJNA856050.

## Author contributions

YC performed most of the investigation, experiment design, data analysis, and revised the manuscript. BT wrote the manuscript. ZZ contributed to interpretation of the data and analysis and revised the manuscript. KY and MC contributed to technical supporting for the human fecal microbiota *in vitro* fermentation. QX and WW were responsible for the project administration and funding acquisition. WH and YG contributed to the draft review and editing. All authors have read and approved the manuscript.

## Funding

This research was funded by Zhejiang National Key Research and Development Program of China, grant number 2022C02041.

## Conflict of interest

The authors declare that the research was conducted in the absence of any commercial or financial relationships that could be construed as a potential conflict of interest.

## Publisher's note

All claims expressed in this article are solely those of the authors and do not necessarily represent those of their affiliated organizations, or those of the publisher, the editors and the reviewers. Any product that may be evaluated in this article, or claim that may be made by its manufacturer, is not guaranteed or endorsed by the publisher.

## Abbreviations

GM: gut microbiota
SCFA: short-chain fatty acid
SDF: water-soluble dietary fiber
IDF: water-insoluble dietary fiber
ACE: angiotensinogen I converting enzyme
HWE: hot water extraction
UE: ultrasonic assisted extraction
SWE: subcritical water extraction
ME: microwave assisted extraction
INU: the control group used inulin as carbon source of *in vitro* simulated intestinal fermentation
TDF: total dietary fiber.
